# Outcomes of coronary artery bypass surgery using modified del Nido cardioplegia in patients with poor ventricular function

**DOI:** 10.1186/s13019-023-02466-0

**Published:** 2023-11-29

**Authors:** Samuel Brown, Kholoud Nassar, Jacob Razzouk, Abishek K. Kashyap, Mitchell Won, Thaer Shehadeh, Reza Salabat, David G. Rabkin, Joshua S. Chung

**Affiliations:** Department of Cardiothoracic Surgery, Loma Linda University Medical Center, Coleman Pavilion, Suite 21121, 11175 Campus Street, Loma Linda, CA 92354 USA

**Keywords:** Coronary artery bypass grafting, Myocardial protection, Low left ventricular ejection fraction, Del Nido cardioplegia

## Abstract

**Background:**

del Nido cardioplegia (DN) has been shown to be safe in adult patients undergoing isolated coronary artery bypass grafting with normal left ventricular ejection fraction. We sought to determine whether it was also safe in adult patients with diminished left ventricular function.

**Methods:**

All patients with preoperative left ventricular ejection fraction ≤ 40% undergoing isolated coronary artery bypass grafting between 1/1/2019 and 7/10/2022 were retrospectively analyzed. Off-pump and beating heart cases were excluded. Patients were divided by surgeon preference between conventional cardioplegia (CCP) and DN. Baseline and intraoperative characteristics and short-term postoperative outcomes were compared.

**Results:**

Six surgeons performed 829 isolated coronary artery bypass operations during the study. Two-hundred seventy-two met study criteria. Three surgeons used exclusively CCP for the duration of the study, two used exclusively DN and one switched from CCP to DN mid-way through. Group totals were: CCP n = 181 and DN n = 91. There were no significant differences in baseline characteristics including mean left ventricular ejection fraction (CCP 32.5 ± 7.4% vs. DN 33.4 ± 7.29%, *p* = 0.939). Other than a significant decrease in bypass time for DN (113.20 ± 37.2 vs. 122.43 ± 34.3 min, *p* = 0.043) there were no intergroup differences in urgency, number of grafts, ischemic time or incidence of blood transfusion. Postoperative outcomes between CCP and DN were similar including incidence of atrial fibrillation (12.2% vs. 8.8%, *p* = 0.403), intensive care length of stay (3.7 ± 2.3 vs. 4.3 ± 3.7 days, *p* = 0.886), total length of stay (5.7 ± 3.7 vs. 6.3 ± 4.4 days, *p* = 0.922) and 30-day mortality (3.85% vs. 1.10%, *p* = 0.205).

**Conclusion:**

Compared to conventional cardioplegia, del Nido cardioplegia provides equivalent short-term outcomes in patients with low left ventricular ejection fraction undergoing isolated coronary artery bypass grafting.

## Background

Critical to all cardiac operations involving a period of ischemic arrest, meticulous myocardial protection is of amplified importance in patients with diminished left ventricular function to facilitate uneventful separation from cardiopulmonary bypass without the need for multiple high-dose inotropes or new mechanical support [1]. The mainstays of myocardial protection in this vulnerable population are a quick and efficient operation to limit the period of ischemia and hypothermia and effective cardioplegia to protect the myocardium during the ischemic interval. While all surgeons aspire to operative efficiency and most use topical and systemic hypothermia, cardioplegia solutions continue to evolve.

Conventional cardioplegia relies on delivery of hyperkalemia to prevent repolarization of cardiac myocytes resulting in a period of depolarized arrest. Hyperkalemia is appealing because it allows for a rapid arrest and a reliable recovery, but it is associated with intracellular sodium and calcium accumulation during the period of arrest [2]. It also requires redosing every fifteen to twenty minutes due to washout of the potassium, thus potentially prolonging the period of myocardial ischemia.

Developed by Pedro del Nido and colleagues at the University of Pittsburgh in the early 1990s for the immature myocardium and modified since, del Nido cardioplegia has expanded to adult populations. Del Nido cardioplegia differs from conventional cardioplegia in several ways: it counteracts the negative effects of hyperkalemia by reducing the potassium concentration and adding the sodium channel blocker Lidocaine which increases the refractory period of the cardiac myocyte [3] and prolongs the period of arrest because it remains in adequate concentrations to continually affect the myocardium. Sodium channel blockade also polarizes the cell membrane to some degree, preventing intracellular sodium and calcium accumulation and allowing for reduction in energy consumption [2, 4, 5]. In addition, magnesium, a natural calcium channel blocker, also reduces the intracellular accumulation of calcium preventing diastolic stiffness from interfering from myocardial recovery. Sodium bicarbonate scavenges excess hydrogen ions which interfere with energy production during periods of anaerobic glycolysis and finally, mannitol is added to scavenge free radicals and reduce myocardial swelling. Del Nido cardioplegia can be delivered as a single dose or re-dosed after 60–90 min, depending on the anticipated length of the operation, therefore improving operative flow and potentially allowing for reduced ischemic time. Since it’s introduction almost thirty years ago modifications to the chemical composition and dosing frequency have occurred and these solutions are often referred to colloquially as ‘del Nido’ formulations while they are technically somewhat different from the original description. We use the term ‘del Nido cardioplegia’ to refer to solutions based on Dr. del Nido’s principles described above.

While del Nido cardioplegia has been shown to be safe during isolated coronary artery bypass grafting in patients with normal left ventricular function [6–9], its use in patients with diminished left ventricular function has received little attention in the literature. In fact, del Nido cardioplegia is avoided by some surgeons in the setting of low left ventricular function due to concern for the adequacy of its myocardial protection despite the potential advantage of reducing the ischemic time. We sought to investigate the safety of del Nido cardioplegia in the setting of low ejection fraction patients undergoing isolated coronary artery bypass surgery.

## Methods

**Fig. 1:**
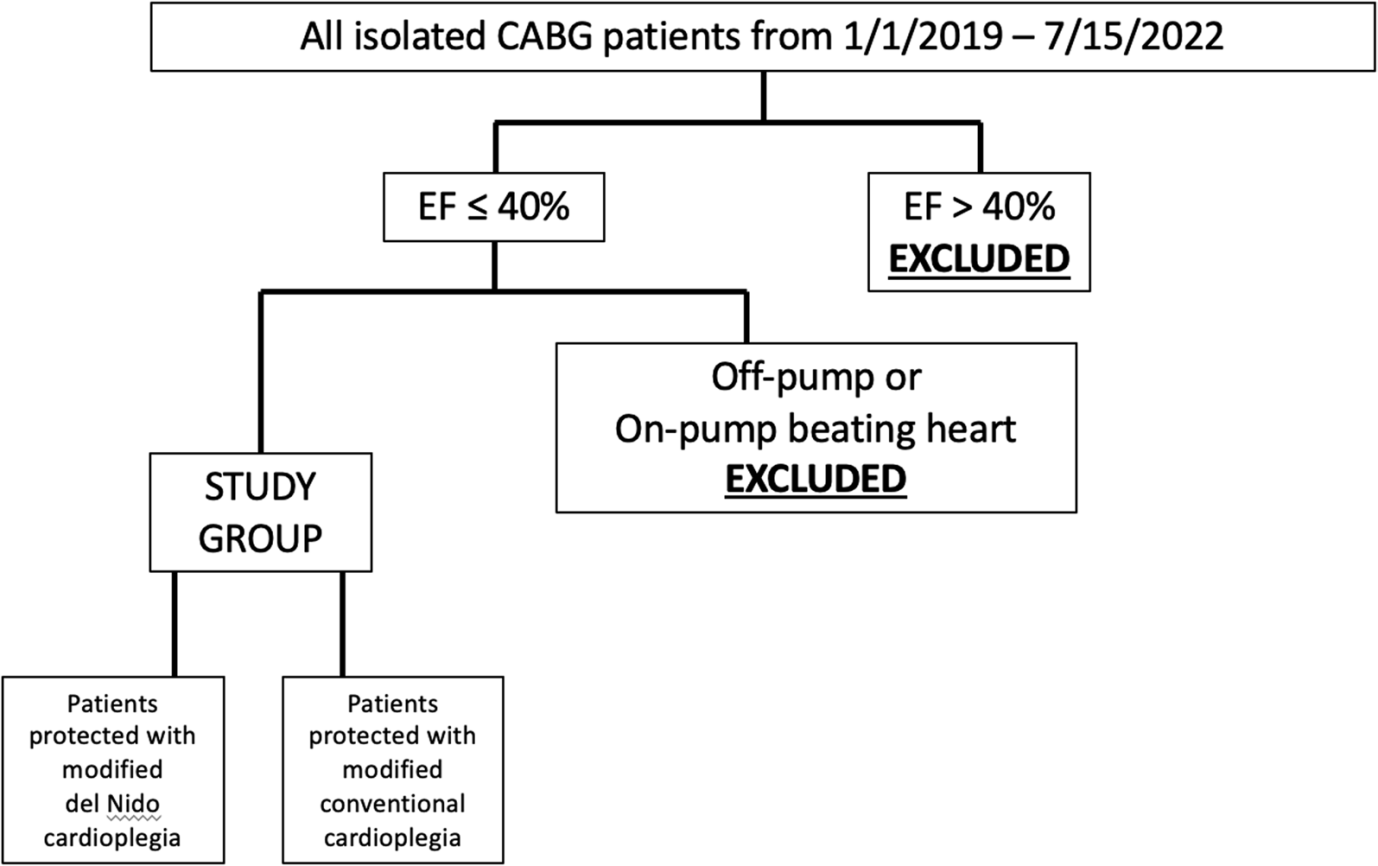
Flow diagram of patient enrollment

After obtaining Institutional Review Board approval the adult cardiac surgery database of Loma Linda University Medical Center was retrospectively reviewed from January 1, 2019 to July 15, 2022. All patients that underwent isolated coronary artery bypass grafting (CABG) were identified and those with a preoperative left ventricular ejection fraction of 40% or less were included in the study. Patients who underwent off-pump or beating heart CABGs were excluded, the remainder were retrospectively divided between those who received modified del Nido cardioplegia and those who received conventional cardioplegia (Fig. 1). Patients’ electronic medical records were reviewed and baseline characteristics including age, gender, weight, body mass index, ethnicity and comorbidities were documented. Preoperative left ventricular ejection fraction was determined from transthoracic or transesophageal echocardiograms performed prior to induction of anesthesia. In emergency cases where preoperative echocardiograms were not performed we used the intra-operative transesophageal echo that was inserted after induction of general anesthesia. We defined the urgency as cases as follows: elective cases came in from home for their previously scheduled operation, urgent cases were patients admitted to the hospital and required operative intervention prior to discharge and emergency cases were those that required surgery as soon as an operating room was available. When a range in left ventricular ejection fraction was documented we used the average of the range. We reviewed operative notes and intraoperative trans-esophageal echocardiograms to document intraoperative characteristics such as the urgency of the case, number of grafts performed, cardiopulmonary bypass time, aortic cross-clamp (ischemic) time and weaning time (difference between the bypass time and the ischemic time) as well as transfusion requirements and changes in post-reperfusion ventricular function. Incidence of postoperative new-onset atrial fibrillation, intensive care unit (ICU) length of stay (LOS), cumulative postoperative LOS and 30-day mortality were determined from the electronic medical record and recorded.

### Surgical technique and myocardial protection

**Fig. 2:**
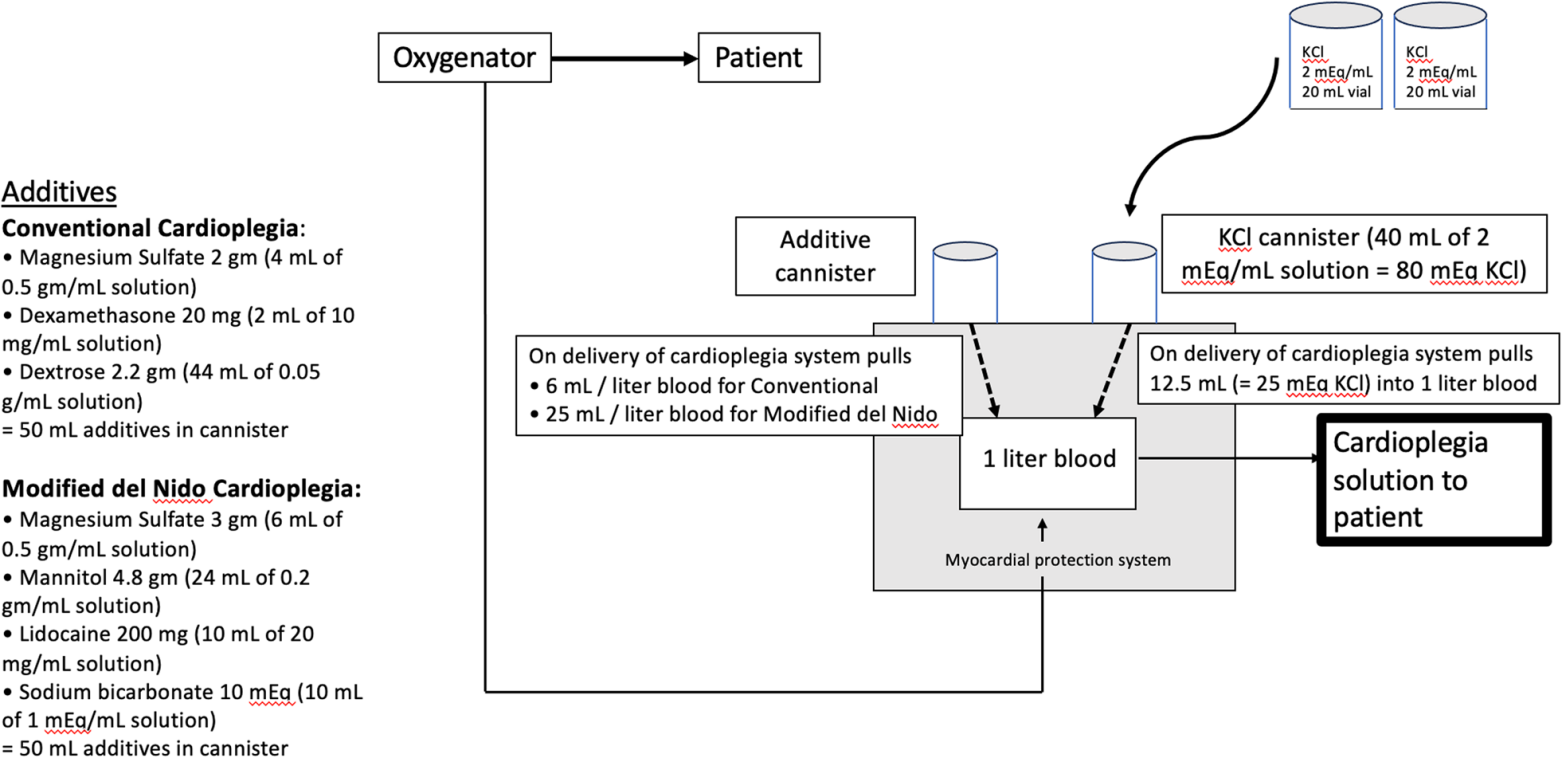
Method of mixing the cardioplegia solutions

All operations were performed through a median sternotomy with central cannulation after the conduits were harvested. Patients were placed on cardiopulmonary bypass and cooled to 32–33 °C. Cardioplegia was delivered at 4 °C using the Quest Myocardial Protection System (Quest Medical, Inc., Allen, Texas) which allows for delivery of oxygenated blood from the oxygenator to the heart after addition of additives which differed depending on which cardioplegia strategy (conventional vs. del Nido) was employed. The type of cardioplegia, conventional vs. del Nido was based on the surgeon’s preference. Our del Nido formulation differs from the classical del Nido composition [4] which used a base solution of Plasma-Lyte A (Baxter Healthcare Corporation, Deerfield, IL) to which the cardioplegic additives were added and then this crystalloid component was mixed with blood in a ratio of four parts crystalloid to one part oxygenated whole blood. Our approach was to add the cardioplegic additives (whether conventional cardioplegia or del Nido) directly to the patient’s whole blood rather than in a crystalloid base (Fig. 2).

Components of additives used in each solution are shown in Table 1 (and Fig. 2).

**Table 1:** Cardioplegia components

Conventional cardioplegia	Modified del Nido cardioplegia
KCl 25 mEq	KCl 25 mEq
Dextrose 2.2 g	Lidocaine 200 mg
Magnesium sulfate 2 g	Magnesium sulfate 3 g
Dexamethasone 20 mg	Mannitol 4.8 g
	Sodium Bicarbonate 10 mEq
6 mL additive in 1 L blood	25 mL additive in 1 L blood

For the conventional cardioplegia 6 mL of additives was added to one liter of patients’ oxygenated blood, for del Nido 25 mL of additives were added to one liter of patients’ oxygenated blood.

Warm induction was used in patients who were actively ischemic. Cardioplegia was delivered in both antegrade and retrograde directions. The amount delivered was based on patients’ weight and surgeon preference but the initial dose was generally around 1500 mL. The rate of delivery for antegrade was 250–400 mL/min keeping the cardiplegia line of pressure in the aortic root around 200 mm Hg. Retrograde cardioplegia was administered at 200–300 mL/minute keeping coronary sinus pressure around 40–45 mm Hg. Mild systemic hypothermia (32–33 °C) and topical hypothermia were used in all cases. Repeat doses of conventional cardioplegia were given retrograde and down the vein grafts every fifteen to twenty minutes. For del Nido repeat doses were planned at 60 min from the initial dose if the anticipated cross-clamp time was greater than 90 min. A warm dose of blood (‘hot shot’) was given through the coronary sinus or aortic root as well as 100 mg lidocaine, 2 gm Magnesium and 12.5 g Mannitol (del Nido only) which were given directly into the pump reservoir at the end of the ischemic interval prior to removing the cross-clamp.

### Follow-up

Patients were admitted to the ICU and down-graded to a telemetry unit when they were weaned from mechanical ventilation, off inotropic and pressor support, and had central venous and arterial lines removed. When standard criteria were met patients were discharged on aspirin, beta blockers and statins. Long-acting calcium channel blockers were also given in patients who had radial artery conduits. All patients were followed up at thirty days with a phone call.

### Statistical analysis

Descriptive statistics were used to summarize demographics and pre- and postoperative variables. If a variable was normally distributed and continuous, the mean ± standard deviation was used; otherwise, median [interquartile range] was used. All the continuous univariate analyses were completed using a 2-sample *t* test or a Wilcoxon rank-sum test depending on if normality assumption was met and the categorical analysis with Chi squared unless the cells with expected counts less than 5 exceeded 20% then Fisher exact test was used. *p* values < 0.05 were considered statistically significant.

## Results

During the study period 829 isolated CABGs were performed by six surgeons. Of these, 272 (32.8%) met inclusion criteria. Of the 272 low left ventricular ejection fraction patients undergoing isolated CABG, 181 were in the conventional cardioplegia group and 91 in the del Nido group. Three of the surgeons used exclusively conventional cardioplegia for the duration of the study period, two surgeons used exclusively del Nido cardioplegia for the duration of the study period and one surgeon switched mid-way through from using exclusively conventional to exclusively del Nido. In no operation did the surgeon convert myocardial protection strategy either from del Nido to conventional cardioplegia or the other way around.

Baseline characteristics are shown in Table 2.

**Table 2:** Comparison of baseline characteristics

Baseline characteristics	Conventional group (n = 181)	Mod Del Nido group (n = 91)	*p* value
Age (years ± SD)	64.2 ± 10.7	63.6 ± 11	0.972
Gender (%)			
Male	82.4	75.8	0.197
Female	17.6	24.2	
Weight (kg ± SD)	84.3 ± 18.1	81.1 ± 17.25	0.935
BMI ± SD	28.5 ± 5.3	27.94 ± 5.4	0.947
Preop EF (mean % ± SD)	32.5 ± 7.4	33.4 ± 7.29	0.939
Ethnicity (#, %)			
White	86 (47.5)	38 (41.8)	0.291
Hispanic	73 (40.3)	31 (34.1)	
Asian	12 (6.6)	15 (16.5)	
Black	5 (2.8)	2 (2.2)	
Other/unknown	5 (2.8)	5 (5.5)	
Comorbidities (#, %)			
Hypertension	165 (91.2)	76 (83.5)	0.061
Diabetes	125 (69.1)	53 (58.2)	0.077
COPD	23 (12.7)	6 (6.6)	0.123
ESRF	30 (16.5)	8 (8.8)	0.081
Prior stroke	15 (8.3)	6 (6.6)	0.621
Prior cardiac surgery	3 (1.7)	1 (1.1)	0.710
Cardiogenic shock (or IABP)	15 (8.29)	11 (12.1)	0.314

*SD* standard deviation, *BMI* body mass index, *preop EF* preoperative left ventricular ejection fraction, *COPD* chronic obstructive pulmonary disease, *ESRF* end-stage renal failure, *IABP* intra-aortic balloon pump

There were no significant differences between groups. Mean preoperative left ventricular ejection fraction for the conventional cardioplegia group was 32.5 ± 7.4% and for the del Nido group it was 33.4 ± 7.29%.

Operative characteristics are shown in Table 3.

**Table 3:** Comparison of intra-operative characteristics

Operative characteristics	Conventional group (n = 181)	Mod Del Nido group (n = 91)	*p* value
Urgency of case (#, %)			
Elective	111 (61.3)	51 (56.0)	0.402
Urgent	55 (30.4)	27 (29.7)	0.903
Emergent	15 (8.3)	12 (13.2)	0.202
Number of grafts (mean ± SD)	3.26 ± 0.8	3.28 ± 0.79	0.845
Bypass time (mean min ± SD)	122.43 ± 34.3	113.20 ± 37.2	**0.043**
Ischemic time (mean min ± SD)	94.55 ± 36.43	89.24 ± 28.73	0.226
Weaning time (mean min ± SD)	27.88 ± 30.15)	23.96 ± 19.5	0.261
Blood transfusion (#, %)	81 (44.8)	37 (40.7)	0.521
Postop hgb (mean g/dL ± SD)	10.5 ± 2.03	11.1 ± 2.0	**0.022**
Improvement in LVEF (mean % ± SD)	7.21 ± 10.39	8.66 ± 8.29	0.248

*SD* standard deviation, *min* minutes, *postop hgb* postoperative hemoglobin, *g* gram, *dL* deciliter, *LVEF* left ventricular ejection fraction. Significant *p* values are rendered in bold

There were no differences in the urgency of the cases. The number of grafts performed did not differ (3.26 ± 0.8 CCP vs. 3.28 ± 0.79 DN, *p* = 0.845). The cardiopulmonary bypass time was significantly shorter for the del Nido group: 113.20 ± 37.2 min vs. 122.43 ± 34.3 min, *p* = 0.043) but the ischemic times and weaning times (difference between cross-clamp and bypass times) were not significantly different.

Outcome characteristics are shown in Table 4.

**Table 4:** Comparison of postoperative outcomes

Outcome characteristics	Conventional group (n = 181)	Mod Del Nido group (n = 91)	*p* value
Postop Afib (#, %)	22 (12.2)	8 (8.8)	0.403
Postop LOS (mean ± SD)	5.7 ± 3.7	6.3 ± 4.4	0.922
ICU LOS (mean ± SD)	3.7 ± 2.3	4.3 ± 3.7	0.886
30-day mortality (%)	3.85	1.10	0.205
Stroke (#, %)	4 (2.2)	2 (2.2)	0.995

*Postop* postoperative, *Afib* atrial fibrillation, *LOS* length of stay, *SD* standard deviation, *ICU* intensive care unit

There were no differences between the two groups for postoperative new-onset atrial fibrillation (12.2% vs. 8.8%, *p* = 0.404), postoperative length of stay (5.7 ± 3.7 vs. 6.3 ± 4.4 days, *p* = 0.922), intensive care unit length of stay (3.7 ± 2.3 vs. 4.3 ± 3.7 days, *p* = 0.886) or 30-day mortality (3.85% vs. 1.10%, *p* = 0.205).

## Discussion

Despite improvements in medical therapy, surgical techniques and perioperative care, patients undergoing isolated CABG with low pre-operative left ventricular ejection fraction remain a challenging population and are at higher risk of postoperative complications [10]. Low left ventricular ejection fraction is common among patients undergoing coronary bypass surgery accounting for a third of the patients undergoing isolated CABG in our study. Various cardioplegia solutions have been used to protect the damaged myocardium during the period of ischemic arrest with whole blood solutions used most commonly in the modern era. As del Nido cardioplegia has gradually migrated from the pediatric to the adult populations evidence is accumulating to support its use but little has been reported in the subset of patients with preoperative left ventricular dysfunction. In this paper we present our experience with del Nido cardioplegia in patients undergoing isolated CABG with poor left ventricular function. Our primary findings were that: (1) there was no difference in 30-day mortality in patients receiving del Nido and conventional cardioplegia and (2) surrogates for the adequacy of intraoperative myocardial protection such as weaning time from bypass, improvements in left ventricular ejection fraction, new onset atrial fibrillation, ICU and hospital length of stay were not different between groups either. Our results support and extend previous work done by Timek and colleagues [11] who showed in a subgroup analysis of 325 propensity-matched patients undergoing isolated CABG resulting in 38 patients in each group all with left ventricular ejection fraction ≤ 35% that del Nido cardioplegia was non-inferior to blood cardioplegia in terms of 30-day mortality and development of atrial fibrillation.

Advantages of del Nido cardioplegia in our study included significantly shorter cardiopulmonary bypass times and trends towards reduced ischemic and weaning times that fell short of statistical significance. Although we did not have differences in the need for blood product transfusion between groups, patients in the del Nido group did have significantly higher postoperative hemoglobin levels as other groups have also reported [12]. This difference, while statistically significant was somewhat subtle (10.5 ± 2.03 g/dL vs. 11.1 ± 2.0 g/dL) likely due to the very small crystalloid component of each solution. Del Nido cardioplegia also provides the convenience of not disrupting the flow of the operation by avoiding or reducing maintenance doses of cardioplegia. Concerns about using del Nido in patients with multivessel coronary disease have been reported, even by programs that use it for other cases [13]. Coronary obstruction can result in inadequate antegrade cardioplegia delivery and potentially poor myocardial protection, which may be more pronounced with longer periods of ischemia. Our group delivered both types of cardioplegia in both antegrade and retrograde directions and down the grafts to avoid this problem.

While the theoretical advantage of shorter bypass times did not result in improvement in measured outcomes, the difference in our study (less than ten minutes) while statistically significant was likely not clinically significant. Compared to other studies [6–9] our bypass times are somewhat longer but this is likely confounded by the fact that our study focused exclusively on patients with low LVEF who often require longer weaning time from bypass. Our bypass times may also have been impacted by our training environment and our emphasis on intraoperative resident education.

## Conclusion

There is growing body of literature that del Nido cardioplegia is safe to use in the adult population, even in higher risk cases such as in patients after acute myocardial infarction, older patients and those with elevated Society for Thoracic Surgery risk scores [11, 14], to which our study adds those patients undergoing isolated CABG with poor left ventricular function. Limitations of our study include its non-randomized and retrospective nature. While not randomized, individual surgeons did not change their approach to myocardial protection based on the patient but rather five of the six surgeons used the same type of cardioplegia for the duration of the study and one surgeon switched mid-way through. In addition, baseline characteristics were not different between the two groups, suggesting that potential confounding due to the non-randomized nature of the study was limited. We also were not able to compare postoperative troponin levels as a marker of the adequacy of myocardial protection because they are not routinely measured at our institution.

## Data Availability

The dataset used during the study are not publicly available for HIPAA compliance purposes but are available from the corresponding author on reasonable request.

## Abbreviations

CABG: Coronary artery bypass grafting
ICU: Intensive care unit
LOS: Length of stay
mL: Milliliters
gm: Gram
